# Intuitionistic fuzzy PAMSSEM method for MAGDM incorporating cumulative prospect theory and its application to the assessment on water resource carrying capacity

**DOI:** 10.1371/journal.pone.0352155

**Published:** 2026-07-10

**Authors:** Chunlan Lv, Guiwu Wei, Qing Feng, Yanli Chen, Qian Huang

**Affiliations:** 1 School of Economics and Management, Yibin University, Yibin, P. R. China; 2 Collaborative Innovation Center for Shipping and Logistics in the Upper Reaches of the Yangtze River, Yibin, P. R. China; 3 School of Business, Sichuan Normal University, Chengdu, P. R. China; 4 School of Management and Economics, University of Electronic Science and Technology of China, Chengdu, P. R. China; 5 College of Management Science, Chengdu University of Technology, Chengdu, P. R. China; Chinese Culture University, TAIWAN

## Abstract

Assessing water resources carrying capacity (WRCC) is essential for regional high-quality development. However, most existing WRCC assessment models fail to handle uncertainties and mixed data arising from multiple criteria, which compromises their practical applicability. To address this limitation, this study integrates cumulative prospect theory (CPT) with the PAMSSEM outranking method to develop a novel intuitionistic fuzzy CPT-PAMSSEM model. Then the proposed method is validated through a case study of four cities in the middle and lower reaches of the Tuojiang River Basin. Results show that: (1) WRCC varies significantly across the four cities: Luzhou and Ziyang show favorable conditions, Zigong is near the critical threshold, and Neijiang faces a severe water resource shortage crisis. (2) the proposed model markedly improves the discrimination of different evaluation results, achieving a differentiation level approximately 3–6 times greater than that of conventional methods. These findings provide actionable insights for sustainable water management.

## 1. Introduction

### 1.1 Background

Water resources are one of the most important natural resources and are a key foundation for maintaining the stability of the economy and society. Driven by economic growth, population expansion, ecological degradation, water pollution, and water depletion, the water resource carrying capacity (WRCC), an extension concept of the sustainable development in the field of water resources, has gradually attracted attention [1,2].

WRCC is defined as “the maximum population size, the level of economic activity, and the urban scale that the water environment can support, for a given system state, region, and period, without compromising the sustainability of the local water environment” [3,4]. WRCC operationalizes coordination between urban socioeconomic processes and the water environment, constituting a primary determinant of urban growth and a cornerstone of human well-being [5]. Therefore, evaluating WRCC is crucial for the sustainable utilization of water resources. Many factors such as urban population, economy, and environment can affect WRCC, particularly the balance between supply and demand, water quality conditions, water ecological environment, and the synergy effect of urban development [6]. Rigorous WRCC assessment is equipped for policymakers to diagnose and reconcile the tensions in regional sustainability transitions.

### 1.2 Literature review

Currently, research on WRCC has been rapidly advancing and attracting growing scholarly attention. Global studies have proposed a range of methodologies to investigate and quantify WRCC from diverse perspectives. Commonly employed approaches include comprehensive indicator evaluations that integrate multiple analytical techniques, such as the extended VIKOR [4], the Entropy Weight Method [7], the combined weighting TOPSIS model [8], the spatiotemporal scale [9,10], based on machine learning [11], and other multidimensional mathematical models [12–15].

The evaluation of WRCC poses a challenge, and the reliance on a single method often entails inherent limitations. Consequently, integrating multiple methodologies to effectively address the shortcomings of single approaches is necessary. For instance, an improved TOPSIS model based on grey relational analysis was applied to comprehensively evaluate the water resources carrying capacity in Gansu Province from 2009 to 2022 [16]; Zhang et al. integrated the Improved Coefficient of Variation Method (ICVM) with the Improved Analytic Hierarchy Process (IAHP) to simultaneously determine subjective and objective weights, thereby enabling a multidimensional assessment of regional water resources carrying capacity [17]; Keyvanfar et al. quantified the relative importance of water resources carrying capacity assessment indicators by integrating the Analytic Hierarchy Process (AHP), entropy method, and CRITIC method, thereby establishing a balanced and objective weight framework [18]; A growing body of research has enhanced the Analytic Hierarchy Process (AHP)-based Accelerated Genetic Algorithm (AGA-AHP) method to optimize the expert evaluation matrix and determine subjective weights for assessing regional water resources carrying capacity [19]; A study has proposed a time-varying Preference Ranking Organization Method for Enrichment Evaluation (PROMETHEE) II model that integrates time series analysis and multi-criteria decision-making (MCDM) to address the dynamic trends in water resources carrying capacity and uncertainties in the assessment process [20]; Zhou et al. proposed an integrated approach for spatio-temporal assessment and attribution of water resources carrying capacity incorporating AHP, TOPSIS, and lorenz asymmetry coefficient methods [21]; Agheli et al. established a hybrid framework combining TOPSIS, the Cloud Model, and Bayesian Networks to improve uncertainty reasoning and evaluation accuracy in WRCC assessments [22].

### 1.3 Research gap and motivation

These studies have achieved significant advancements in assessing WRCC, thereby giving a robust foundation for the development of related fields. Nevertheless, certain aspects still require further research. Firstly, most existing evaluation models are based on compensatory assumptions, which do not adequately consider the non-replaceability of indicators or the existence of critical thresholds. As a result, the actual carrying capacity of cities with significant weaknesses may be overestimated. Secondly, traditional data processing techniques such as standardization or weighted summation, often lead to partial loss of original information, rendering the assessment results overly abstract and generalized. Finally, conventional methods exhibit limitations in handling mixed data types, and are not well-suited to effectively process the fuzzy and qualitative information in real-world scenarios.

PAMSSEM (Procedure d’Agrégation Multicritère de type Surclassement de Synthèse pour Évaluation Mixte), a hybrid multi-criteria decision-making method, integrates the structural equation model (SEM) with the preference analysis model (PAM) to address evaluation challenges arising from data heterogeneity and missing information [23,24]. By incorporating three key thresholds (indifference threshold q, preference threshold p, and veto threshold υ), PAMSSEM calibrates the decision-maker’s sensitivity to variations between options. This allows the model to produce tailored rankings that reflect subtle preferences, with trade-offs between criteria meticulously governed by strictly enforced non-compensatory rules [25]. The urban water resource carrying capacity represents a typical natural-social composite system assessment challenge. The PAMSSEM method effectively addresses the multi-dimensional and nonlinear characteristics of water resource systems. It achieves this by leveraging its non-compensatory super-classification logic and flexible preference threshold system, with particular efficacy in tackling the systems’ susceptibility to critical shortcomings. This enables the more comprehensive and robust decision-making support.

However, there are certain issues with the traditional PAMSSEM approach when it comes to making useful decisions. On the one hand, the inherent ambiguity of human judgments and the complexity of real-world issues make it difficult or impossible to get correct evaluation values in the process of evaluating the WRCC [26]. The concept of Intuitionistic Fuzzy (IF) sets was first introduced by Atanassov in 1986 [27], and has since been successfully applied across a wide range of decision-making contexts [28–33]. On the other hand, Cumulative prospect theory (CPT), an upgraded version of prospect theory (PT) [34], can incorporate DMs’ psychological senses into their decision-making behaviors while successfully capturing DMs’ psychological actions in the face of benefits and losses [35]. Combining CPT with some MADM approaches to cope with uncertain circumstances is currently an attraction in decision-making research [36–38]. However, there has been limited research on the PAMSSEM approach for addressing uncertain problems. Notably, the CPT-PAMSSEM technique was not developed based on IFSs. Based on this, the primary goal of this study is to extend the unique PAMSSEM approach to IFSs and apply this combined method to the evaluation of WRCC, which can more completely account for the limited rationality of decision-makers’ thinking.

### 1.4 Contribution and novelty

The proposed IF-CPT-PAMSSEM method addresses several key limitations of existing MCDM techniques. As presented in Table 1, the method is systematically compared with the established IF-CPT-MABAC, IF-CPT-VIKOR and IF-CPT-TODIM approaches in terms of theoretical framework, computational efficiency, decision quality, and practical applicability.

**Table 1:** A comparative analysis of IF-CPT-PAMSSEM with existing methodologies.

	IF-CPT-PAMSSEM	IF-CPT-MABAC	IF-CPT-VIKOR	IF-CPT-TODIM
Theoretical framework	Accurately and reliably reflects the relative performance of the schemes	Sensitive to outliers	Suppress plans with distinctive features or critical flaws	Model parameters are highly sensitive
Computational efficiency	Highly structured and systematic	Highest computational efficiency	High computational efficiency	Lowest computational efficiency
Decision quality	High robustness; excellent discrimination; strong security	Boundary classification using positive and negative distances may cause discontinuous results and reduced stability	It may not be the globally optimal solution.	Results show limited stability, suggesting sensitivity to input variations or parameter settings
Practical applicability	Suitable for complex, heterogeneous, multi-source decision-making problems	Well suited for “filtering-type” decisions involving homogeneous information, clear rules, and distinct alternatives	In group decisions with clear interest conflicts, the “compromise” may not satisfy all parties	Highly dependent on decision-makers’ risk preferences

## 2. Methodology

PAMSSEM is a method for ranking schemes based on preference with numerous advantages [25]. However, in classical PAMSSEM, the weight of each attribute is expressed as objective probability, and the perceived value does not correspond to the CPT value function. This study advances the PAMSSEM approach by incorporating CPT’s value and weight functions, plus an attribute weight calculation method, to characterize DMs’ gain-loss decision-making psychology [35]. Besides, it concentrates on an environment of fuzzy intuition, which implies that all data is provided as intuitive fuzzy integers [39–44].

We assume that decision maker is $DM=\left\{DM_{\text{1}},\ldots ,DM_{k}\right\}$, and the weight of decision maker is

**Fig 1:**
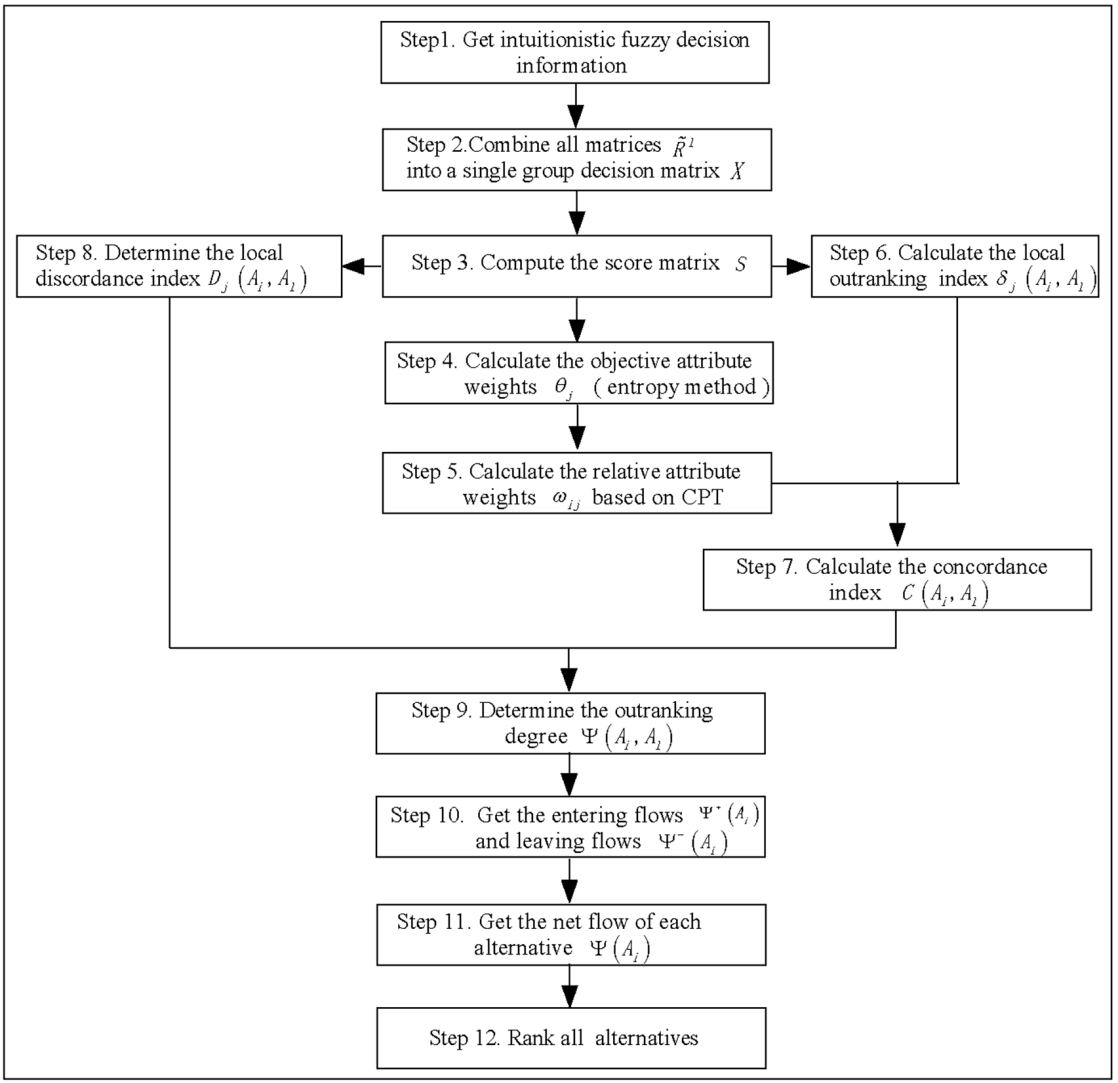
Flowchart of the proposed IF-CPT-PAMSSEM methodology.

$\zeta ={\left(\zeta_{\text{1}},\ldots ,\zeta_{k}\right)}^{T}$. Let $A=\left\{A_{\text{1}},\ldots ,A_{m}\right\}$ is a finite set of alternatives, and $C=\left\{C_{\text{1}},\ldots ,C_{n}\right\}$ is a set of evaluation attributes of the alternatives. The intuitionistic fuzzy decision matrix of the *kth* DM is $R^{\left(k\right)}={\left(\overset{k}{\underset{ij}{r}}\right)}_{m\times n}={\left(\langle \overset{\left(k\right)}{\underset{ij}{\mu }},\overset{\left(k\right)}{\underset{ij}{\upsilon }}\rangle \right)}_{m\times n}$. The steps of the proposed IF-CPT-PAMSSEM methodology are as follows, shown as Fig 1.

**Step 1**. Ensure consistency between benefit and cost attributes.


$$\begin{array}{c} {\tilde{R}}^{\left(k\right)}={\left(\overset{k}{\underset{ij}{\tilde{r}}}\right)}_{m\times n};\;i=1,2,\ldots ,m;j=1,2,\ldots ,n \end{array}$$ (1)

$$\begin{array}{c} \overset{k}{\underset{ij}{\tilde{r}}}=\langle \overset{\left(k\right)}{\underset{ij}{\tilde{\mu }}},\overset{\left(k\right)}{\underset{ij}{\tilde{\upsilon }}}\rangle =\left\{ \begin{array}{c} \langle \overset{\left(k\right)}{\underset{ij}{\mu }},\overset{\left(k\right)}{\underset{ij}{\upsilon }}\rangle ,c_{j}\text{ is positive}\\ \langle \overset{\left(k\right)}{\underset{ij}{\upsilon }},\overset{\left(k\right)}{\underset{ij}{\mu }}\rangle ,c_{j}\text{ is negative} \end{array}\right. \end{array}$$ (2)

**Step 2**. Integrate all matrices ${\tilde{R}}^{\left(k\right)}={\left(\overset{k}{\underset{ij}{\tilde{r}}}\right)}_{m\times n}$ into one group decision matrix $X={\left(x_{ij}\right)}_{m\times n}$ for combining the views of different decision makers.


$$\begin{array}{c} \begin{array}{c} x_{ij}=\langle \mu_{ij},\upsilon_{ij}\rangle =IFWA_{\zeta }\left(\overset{\left(1\right)}{\underset{ij}{\tilde{r}}},\ldots ,\overset{\left(k\right)}{\underset{ij}{\tilde{r}}}\right)\\ =\zeta_{\text{1}}\overset{\left(1\right)}{\underset{ij}{\tilde{r}}}⊕\ldots ⊕\zeta_{k}\overset{\left(k\right)}{\underset{ij}{\tilde{r}}}=\langle 1-\prod_{k=1}^{K}{\left(1-\overset{\left(k\right)}{\underset{ij}{\tilde{\mu }}}\right)}^{\zeta_{k}},\prod_{k=1}^{K}\left(\overset{\left(k\right)}{\underset{ij}{\tilde{\upsilon }}}\right)\rangle \end{array} \end{array}$$ (3)

**Step 3**. The score matrix $S={\left(s_{ij}\right)}_{m\times n}$ can be determined as follows.


$$\begin{array}{c} s_{ij}=\left(\mu_{ij}-\upsilon_{ij}\right)+\pi_{ij}\left(\mu_{ij}-\upsilon_{ij}\right) \end{array}$$ (4)

Here, $\pi_{ij}$ is the hesitancy degree (or uncertainty degree), indicating the degree of uncertainty about the element’s belonging, and $\pi_{ij}=1-\mu_{ij}-\upsilon_{ij}$

**Step 4**. Using the information from the group decision matrix $X={\left(x_{ij}\right)}_{m\times n}$, we can find the objective attribute weights $\theta_{j}$ using the entropy method [45], just as the Eqs. (5) and (6).

Shannon information entropy:


$$\begin{array}{c} E_{j}=-\frac{\text{1}}{lnm}\overset{m}{\underset{i=1}{\sum }}\eta_{ij}\cdot ln\left(\eta_{ij}\right);0\leq E_{j}\leq 1 \end{array}$$ (5)

Where the proportion $\eta_{ij}=\frac{\left|s_{ij}\right|}{\overset{m}{\underset{i=1}{\sum }}\left|s_{ij}\right|}$

Objective attribute weights:


$$\begin{array}{c} \theta_{j}=\frac{1-E_{j}}{\overset{n}{\underset{j=1}{\sum }}\left(1-E_{j}\right)} \end{array}$$ (6)

The entropy technique is an objective approach for determining weights that determines each attribute’s weight based on its observed value. This approach of determining weights successfully eliminates unclear components, allowing us to acquire accurate initial weights.

**Step 5**. The relative attribute weights $\omega_{ij}$ can be derived based on CPT.

Under different circumstances, the cumulative prospect function $V\left(x_{j}\right)$ consists of the value function $v\left(x_{j}\right)$ and the weighting function $w\left(\theta_{j}\right)$, as shown in Eq. (7).


$$\begin{array}{c} V\left(x_{j}\right)=\sum_{j=1}^{m}v\left(x_{j}\right)\cdot w\left(\theta_{j}\right) \end{array}$$ (7)

The value function $v\left(x_{j}\right)$ represents DM’ subjective assessment of income $x_{j}$ compared to the datum point $\overset{\text{0}}{\underset{j}{x}}$. Furthermore, the parameters α and β (the risk attitude of DMs) respectively indicate the preference degrees in the region of gain and loss. Parameter λ denotes the coefficient of loss aversion that is more sensitive to loss than gain [34,35]. A risk-averse DM prioritizes gains and prefers a strategy with better confidence (α≥β, λ>1). In general, α=β=0.88, λ=2.25 [35], and they have been recognized by most scholars [29,38,46]. The value function is determined using Eq. (8).


$$\begin{array}{c} v\left(x_{j}\right)=\left\{ \begin{array}{c} {\left(x_{j}-\overset{\text{0}}{\underset{j}{x}}\right)}^{\alpha },x_{j}\geq \overset{\text{0}}{\underset{j}{x}}\\ -\lambda {\left(\overset{\text{0}}{\underset{j}{x}}-x_{j}\right)}^{\beta },x_{j}<\overset{\text{0}}{\underset{j}{x}} \end{array}\right. \end{array}$$ (8)

The weighting function $w\left(p_{j}\right)$ is validated using the relative magnitude of income $x_{j}$ and the standard point $\overset{\text{0}}{\underset{j}{x}}$. It is formulated by Eq. (9):


$$\begin{array}{c} w\left(\theta_{j}\right)=\left\{ \begin{array}{c} \frac{\overset{\tau }{\underset{j}{\theta }}}{{\left(\overset{\tau }{\underset{j}{\theta }}+{\left(1-\theta_{j}\right)}^{\tau }\right)}^{\frac{\text{1}}{\tau }}},x_{j}\geq \overset{\text{0}}{\underset{j}{x}}\\ \frac{\overset{\sigma }{\underset{j}{\theta }}}{{\left(\overset{\sigma }{\underset{j}{\theta }}+{\left(1-\theta_{j}\right)}^{\sigma }\right)}^{\frac{\text{1}}{\sigma }}},x_{j}<\overset{\text{0}}{\underset{j}{x}} \end{array}\right. \end{array}$$ (9)

Where τ, σ express the curvature for probability weighting function of gain and loss and reflect DMs’ different risk attitudes toward gain and loss [34,35]. In general, τ=0.61,σ=0.69 [35,38,46]. Therefore, the relative attribute weights $\omega_{ij}$ based on CPT is described by Eq. (10):


$$\begin{array}{c} \omega_{ij}=\left\{ \begin{array}{c} \frac{\overset{\tau }{\underset{j}{\theta }}}{{\left(\overset{\tau }{\underset{j}{\theta }}+{\left(1-\theta_{j}\right)}^{\tau }\right)}^{\frac{\text{1}}{\tau }}},s_{ij}\geq \overset{\text{0}}{\underset{j}{s}}\\ \frac{\overset{\sigma }{\underset{j}{\theta }}}{{\left(\overset{\sigma }{\underset{j}{\theta }}+{\left(1-\theta_{j}\right)}^{\sigma }\right)}^{\frac{\text{1}}{\sigma }}},s_{ij}<\overset{\text{0}}{\underset{j}{s}} \end{array}\right. \end{array}$$ (10)

**Step 6.** The local outranking index $\delta_{j}\left(A_{i},A_{l}\right)$ can be calculated using the following expression:


$$\begin{array}{c} \delta_{j}\left(A_{i},A_{l}\right)=\sum_{s_{lj}}\left(\sum_{s_{ij}}{\overset{―}{\delta }}_{j}\left(s_{ij},s_{lj}\right)\cdot f_{ij}\left(s_{ij}\right)\right)\cdot f_{lj}\left(s_{lj}\right);\;i,l\in \left\{1,\ldots ,m\right\},j=1,\ldots ,n \end{array}$$ (11)

There, $s_{ij}$ and $s_{lj}$ are the score functions, and $\int_{\text{-}\infty }^{\text{+}\infty }f_{ij}\left(s_{ij}\right)ds_{ij}=\int_{\text{-}\infty }^{\text{+}\infty }f_{lj}\left(s_{lj}\right)ds_{lj}=1$. Then, the index ${\overset{―}{\delta }}_{j}\left(s_{ij},s_{lj}\right)$ is calculated according to Eq. (12).


$$\begin{array}{c} {\overset{―}{\delta }}_{j}\left(s_{ij},s_{lj}\right)=\left\{ \begin{array}{c} \text{1},\Delta_{j}\left(\text{i,l}\right)\geq -q_{j}\\ \frac{\Delta_{j}+p_{j}}{p_{j}-q_{j}},-p_{j}\leq \Delta_{j}<-q_{j};p_{j}\geq q_{j}\geq 0\\ 0,\Delta_{j}\leq -p_{j} \end{array}\right. \end{array}$$ (12)

There, $\Delta_{j}=s_{ij}-s_{lj}$, $p_{j}$ is the preference threshold, $q_{j}$ is the indifference threshold, and ${\overset{―}{\delta }}_{j}\left(s_{ij},s_{lj}\right)$ can be acquired by Eq. (13).


$$\begin{array}{c} {\overset{―}{\delta }}_{j}\left(s_{ij},s_{lj}\right)=\left\{ \begin{array}{c} \text{1},\Delta_{j}\geq \text{0}\\ \frac{\text{1}}{\text{2}},-\text{1}\leq \Delta_{j}<\text{0};p_{j}\geq q_{j}\geq 0\\ 0,\Delta_{j}<-\text{1} \end{array}\right. \end{array}$$ (13)

**Step 7**. To determine the concordance index $C\left(A_{i},A_{l}\right)$, conduct these steps:


$$\begin{array}{c} C\left(A_{i},A_{l}\right)=\sum_{j=1}^{n}\omega_{ij}\delta_{j}\left(A_{i},A_{l}\right) \end{array}$$ (14)

There, $\omega_{ij}$ are the relative attribute weights.

**Step 8**. Calculate the local discordance index $D_{j}\left(A_{i},A_{l}\right)$:


$$\begin{array}{c} D_{j}\left(A_{i},A_{l}\right)=\sum_{A_{i}}\left(\sum_{A_{l}}{\overset{―}{D}}_{j}\left(s_{ij},s_{lj}\right)\cdot f_{lj}\left(s_{lj}\right)\right)\cdot f_{ij}\left(s_{ij}\right);i,l\in \left\{1,2,\ldots ,m\right\},j=1,\ldots ,n \end{array}$$ (15)

${\overset{―}{D}}_{j}\left(s_{ij},s_{lj}\right)$ can be calculated with Eq. (16).


$$\begin{array}{c} {\overset{―}{D}}_{j}\left(s_{ij},s_{lj}\right)=\left\{ \begin{array}{c} \text{0},\Delta_{j}\geq -p_{j}\\ -\frac{\Delta_{j}+p_{j}}{v_{j}-p_{j}},-v_{j}\leq \Delta_{j}<-p_{j};v_{j}\geq p_{j}\geq q_{j}\geq 0\\ 1,\Delta_{j}\leq -v_{j} \end{array}\right. \end{array}$$ (16)

There, $v_{j}$ is the veto threshold, and it can be determined by the DM.

**Step 9**. Determine the outranking degree $\Psi \left(A_{i},A_{l}\right)$ as follows:


$$\begin{array}{c} \Psi \left(A_{i},A_{l}\right)=C\left(A_{i},A_{l}\right)\cdot \prod_{j=1}^{n}\left[1-\overset{\text{3}}{\underset{j}{D}}\left(A_{i},A_{l}\right)\right];0\leq \Psi \left(A_{i},A_{l}\right)\leq 1 \end{array}$$ (17)

If $\Psi \left(A_{i},A_{l}\right)$ is approximately equal to 1, it indicates that $A_{i}$ is globally superior to $A_{l}$.

**Step 10**. The entering flows $\Psi^{\text{+}}\left(A_{i}\right)$ and leaving flows $\Psi^{\text{-}}\left(A_{i}\right)$ can be determined:


$$\begin{array}{c} \Psi^{\text{+}}\left(A_{i}\right)\text{=}\sum_{l\neq i}\Psi \left(A_{i},A_{l}\right);i,l\in \left\{1,\ldots ,m\right\} \end{array}$$ (18)

$$\begin{array}{c} \Psi^{\text{-}}\left(A_{i}\right)\text{=}\sum_{l\neq i}\Psi \left(A_{l},A_{i}\right);i,l\in \left\{1,\ldots ,m\right\} \end{array}$$ (19)

**Step 11**. Determine the net flow of each alternative $\Psi \left(A_{i}\right)$:


$$\begin{array}{c} \Psi \left(A_{i}\right)\text{=}\Psi^{\text{+}}\left(A_{i}\right)\text{-}\Psi^{\text{-}}\left(A_{i}\right);\;i=1,\ldots ,m \end{array}$$ (20)

**Step 12**. All alternatives are ranked based on $\Psi \left(A_{i}\right)$.

## 3. Case study

In this section, a case is used to explain the decision-making procedure and legitimacy of the proposed IF-CPT-PAMSSEM approach. Moreover, sensitivity and comparison analysis are performed to demonstrate its efficacy.

### 3.1 Study area and data

The Tuojiang River Basin, located in central Sichuan Province, China, serves as an upstream tributary of the Yangtze River. The runoff in the basin is mainly from precipitation, supplemented by water from the Minjiang River via the Dujiangyan Irrigation System. It has a total water resource of $103.4\times 10^{\text{8}}m^{\text{3}}$. But the per capita water resource in the Tuojiang River Basin of Sichuan is only 559 cubic meters, less than a quarter of the average across the Yangtze River Basin, making it a relatively water-scarce area within the Yangtze River Basin. Moreover, the Tuojiang River Basin constitutes a core industrial hub in Sichuan Province, hosting approximately 1,000 major and medium-sized chemical enterprises. Consequently, substantial volumes of industrial wastewater are discharged into the river. Water quality monitoring data and scientific studies consistently indicate degraded water conditions, revealing that the basin’s water quality remains critically poor [47].

Four representative cities along the Tuojiang River Basin—Ziyang, Neijiang, Zigong, and Luzhou—exemplify distinct types of water resource challenges: engineering water scarcity coupled with agriculture-dominated use in Ziyang, extreme physical water shortage in Neijiang, combined engineering and quality-induced scarcity in Zigong, and water quality and ecological constraints in Luzhou. They form a complete and typical socio-hydrological system in the middle and lower reaches of the Tuojiang River. An in-depth analysis of the WRCC of these four cities provides critical insights and direct policy support for the implementation of the Yangtze River Protection Strategy.

The WRCC in this study was comprehensively evaluated from four aspects: water resources, economy, society, and ecological environment, spanning from 2015 to 2024. The water resource data were from the Sichuan Provincial Water Resources Bulletin (http://www.schwr.com/article/category/szygb). While data on ecological environment, social, and economic factors were derived from the Sichuan Statistical Yearbook (http://tjj.sc.gov.cn/scstjj/c105855/nj.shtml) and the Sichuan Water Resources Bulletin. In addition, some index data were obtained by corresponding calculation. For instance, per capita water resources were the total amount of regional water resources divided by the resident population at the end of the year. For missing indicator data in certain years, this study used linear interpolation to fill in the missing data.

### 3.2 Results of IF-CPT-PAMSSEM method

**Fig 2:**
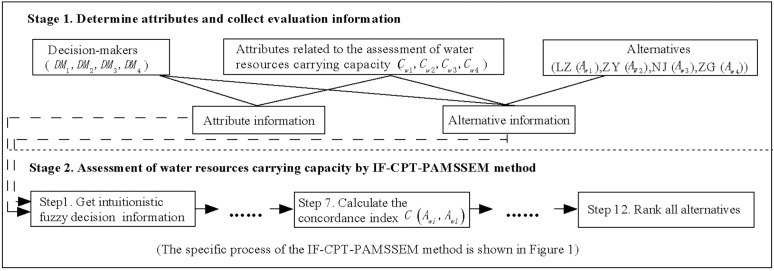
A decision framework for assessing WRCC.

The WRCC system is characterized by dynamic interactions between water resources, economy, society, and ecological environment, which together determine the overall level of WRCC. This study proposes the IF-CPT-PAMSSEM method as a novel approach to analyze the assessment of WRCC. The objective is to evaluate the sustainable utilization potential of urban water resources, identify associated development risks, and provide a scientifically grounded basis for policy formulation in urban planning and water resource management. As shown in Fig 2, the framework is composed of two different stages.

**Stage 1. Determine attributes and collect evaluation information**

**Fig 3:**
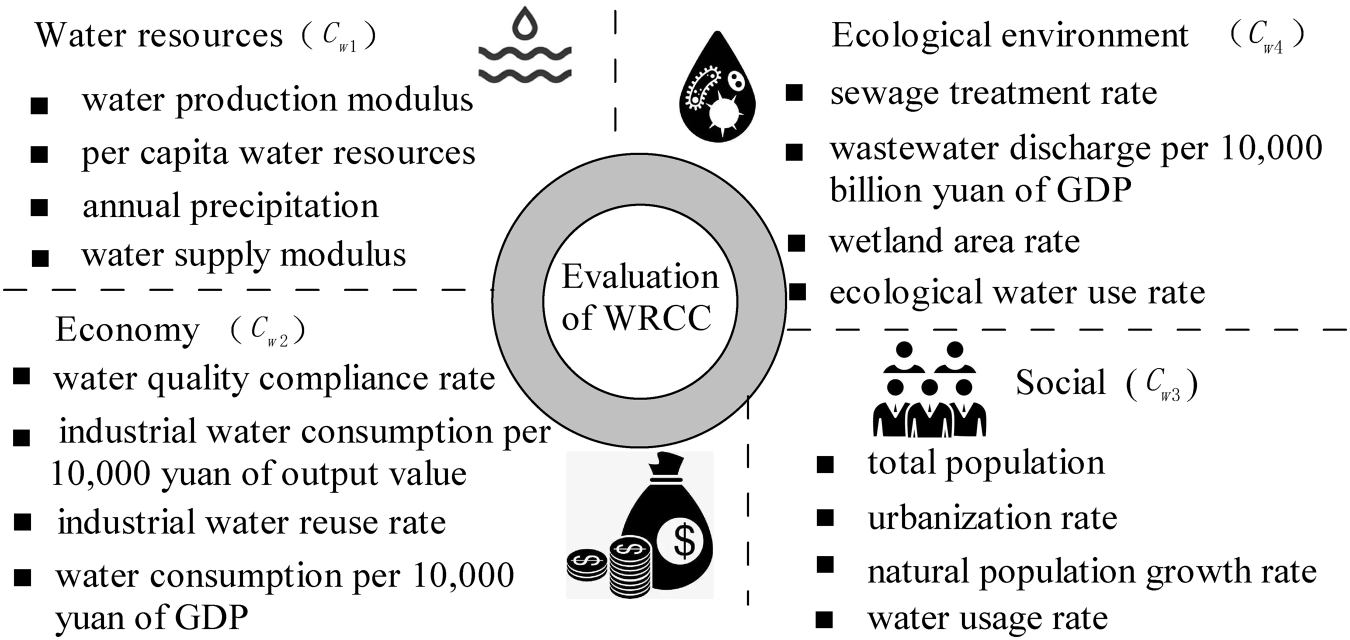
Evaluation criteria for WRCC assessment.

In this study, assuming that four DMs ($DM_{\text{1}},DM_{\text{2}},DM_{\text{3}},DM_{\text{4}}$) participate in the evaluation of WRCC. Weights of four DMs are 0.25, 0.25, 0.25, and 0.25, respectively. Then the WRCC of four cities (Luzhou($A_{w1}$), Ziyang($A_{W2}$), Neijiang($A_{w3}$), Zigong($A_{w4}$)) is ranked. The evaluation criteria can be water resources ($C_{w1}$), economy ($C_{w\text{2}}$), society ($C_{w\text{3}}$) and the ecological environment ($C_{w\text{4}}$), as shown in Fig 3. All criteria are benefit oriented, and detailed descriptions of these characteristics are provided below.

(1) Water resources ($C_{w1}$). Key indicators include water production modulus, per capita water resources, annual precipitation, and water supply modulus [16,48,49].
(2) Economy ($C_{w\text{2}}$). Key indicators include the water quality compliance rate, industrial water consumption per 10,000 yuan of output value, the proportion of water-saving irrigation area, water consumption per 10,000 yuan of GDP, agricultural water consumption per 10,000 yuan of output value, and the industrial water reuse rate [16,48,49].
(3) Society ($C_{w\text{3}}$). Total population; urbanization rate, reflecting urban water resource pressure; natural population growth rate, reflecting water resource pressure caused by population growth; water usage rate, reflecting the comprehensive efficiency of water resource utilization [16,48,49].
(4) Ecological environment ($C_{w\text{4}}$). Key indicators include the centralized sewage treatment rate, reflecting wastewater treatment capacity; wastewater discharge per 10,000 billion yuan of GDP, indicating the pollution level of the water environment caused by economic development; the wetland area rate, reflecting the capacity for natural water replenishment; and the, reflecting ecosystem demand for water resources [16,48,49].

**Stage 2. Assessment of WRCC by IF-CPT-PAMSSEM method**

Through discussions with four invited experts in the field of water resource management, intuitive fuzzy numbers (IFNs) are directly assigned to each linguistic concept according to their professional judgment (Table 2), ensuring that the mapping aligns with the experts’ domain-specific intuitions and practical experiences. The evaluation decision matrices from $DM_{\text{1}},DM_{\text{2}},DM_{\text{3}},DM_{\text{4}}$ are given in supplementary materials, and Step 1 is completed.

**Table 2:** A table comparing linguistic terms to IFNs.

Linguistic terms	IFNs
Perfectly poor	(0.10,0.88)
Quite poor	(0.25,0.70)
Poor	(0.35,0.55)
Medium	(0.50,0.50)
Great	(0.60,0.30)
Quite great	(0.75,0.20)
Perfectly great	(0.90,0.08)

Step 2. Using Eq. (3) to combine all matrices ${\tilde{R}}^{\left(k\right)}={\left(\overset{k}{\underset{ij}{r}}\right)}_{m\times n}$ into a single group decision matrix $X={\left(x_{ij}\right)}_{m\times n}$ (Table 3) for combining the four DMs.

**Table 3:** Group decision matrix $X={\left(x_{ij}\right)}_{m\times n}$.

	$C_{w1}$	$C_{w2}$	$C_{w3}$	$C_{w4}$
$A_{w\text{1}}$	(0.78,0.18)	(0.68,0.25)	(0.67,0.28)	(0.49,0.45)
$A_{w\text{2}}$	(0.82,0.14)	(0.62,0.31)	(0.64,0.29)	(0.49,0.45)
$A_{w\text{3}}$	(0.68,0.25)	(0.64,0.29)	(0.62,0.31)	(0.44,0.49)
$A_{w\text{4}}$	(0.80,0.16)	(0.53,0.44)	(0.75,0.19)	(0.49,0.45)

Step 3. Compute the score matrix $S={\left(s_{ij}\right)}_{m\times n}$ through Eq. (4).


$$\begin{array}{c} S={\left(s_{ij}\right)}_{m\times n}\text{=}\left[\begin{array}{c} \text{0.63 0.47 0.41 0.05}\\ \text{0.71 0.34 0.38 0.05}\\ \text{0.47 0.37 0.34 0.05}\\ \text{0.67 0.09 0.59 0.05} \end{array}\right] \end{array}$$ (21)

Step 4. Calculate the objective attribute weights $\theta_{j}=\left\{\text{0.07,0.77,0.15,0.01}\right\}$ by using Eqs. (5) and (6).

Step 5. Calculate the relative attribute weights $\omega_{ij}$ (Table 4) based on CPT using Eqs. (7)–(10).

**Table 4:** The relative attribute weights $\omega_{ij}$.

	$C_{w1}$	$C_{w2}$	$C_{w3}$	$C_{w4}$
$\omega_{\text{1}j}$	0.16	0.58	0.21	0.048
$\omega_{\text{2}j}$	0.16	0.58	0.21	0.048
$\omega_{\text{3}j}$	0.14	0.60	0.22	0.035
$\omega_{\text{4}j}$	0.15	0.59	0.21	0.045

Step 6. Calculate the local outranking index $\delta_{j}\left(A_{wi},A_{wl}\right)$ by Eqs. (11)–(13), and four outranking matrices can be obtained:

Outranking Matrix for Criterion 1 ($C_{w1}$)


$$\begin{array}{c} \delta_{1}(A_{w},A_{w})=[\begin{array}{cccc} - & 0.42 & 1 & 1\\ 1 & - & 1 & 1\\ 0 & 0 & - & 0\\ 1 & 1 & 1 & - \end{array}] \end{array}$$ (22)

Outranking Matrix for Criterion 2 ($C_{w\text{2}}$)


$$\begin{array}{c} \delta_{\text{2}}\left(A_{wi},A_{wl}\right)\text{=}[\begin{array}{cccc} - & 1 & 1 & 1\\ 0 & - & 1 & 1\\ 0.03 & 1 & - & 1\\ 0 & 0 & 0 & - \end{array}] \end{array}$$ (23)

Outranking Matrix for Criterion 3 ($C_{w\text{3}}$)


$$\begin{array}{c} \delta_{\text{3}}\left(A_{wi},A_{wl}\right)\text{=}[\begin{array}{cccc} - & 1 & 1 & 0\\ 1 & - & 1 & 0\\ 0.5 & 7 & 1- & 0\\ 1 & 1 & 1 & - \end{array}] \end{array}$$ (24)

Outranking Matrix for Criterion 4 ($C_{w\text{4}}$)


$$\begin{array}{c} \delta_{\text{4}}\left(A_{wi},A_{wl}\right)\text{=}[\begin{array}{cccc} - & 1 & 1 & 1\\ 1 & - & 1 & 1\\ 0.02 & 0.02 & - & 0.02\\ 1 & 1 & 1 & - \end{array}] \end{array}$$ (25)

Step 7. Calculate the concordance index $C\left(A_{wi},A_{wl}\right)$ by Eq. (14), and Table 5 indicates the indifference threshold q, the preference threshold p and the veto threshold v of each attribute, and we can get:

**Table 5:** Parameter values of single sample study.

*Parameter*	$C_{w1}$	$C_{w2}$	$C_{w3}$	$C_{w4}$
q	0.05	0.05	0.05	0.05
p	0.10	0.10	0.10	0.10
v	0.15	0.15	0.15	0.15


$$\begin{array}{c} C\left(A_{wi},A_{wl}\right)=[\begin{array}{cccc} - & 0.91 & 10. & 79\\ 0.42 & - & 1 & 0.79\\ 0.14 & 0.82 & - & 0.60\\ 0.40 & 0.40 & 0.40 & - \end{array}] \end{array}$$ (26)

Step 8. Determine the local discordance index $D_{j}\left(A_{wi},A_{wl}\right)$ by Eqs. (15) and (16). This yields four discordance matrices:

Discordance Matrix for Criterion 1 ($C_{w1}$)


$$\begin{array}{c} D_{\text{1}}\left(A_{wi},A_{wl}\right)=[\begin{array}{cccc} - & 0 & 0 & 0\\ 0 & - & 0 & 0\\ 1 & 1 & -1 & \\ 0 & 0 & 0 & - \end{array}] \end{array}$$ (27)

Discordance Matrix for Criterion 2 ($C_{w\text{2}}$)


$$\begin{array}{c} D_{\text{2}}\left(A_{wi},A_{wl}\right)[\begin{array}{cccc} - & 0 & 0 & 0\\ 0.65 & - & 0 & 0\\ 0 & 0 & - & 0\\ 1 & 1 & 1 & - \end{array}] \end{array}$$ (28)

Discordance Matrix for Criterion 3 ($C_{w\text{3}}$)


$$\begin{array}{c} D_{\text{3}}\left(A_{wi},A_{wl}\right)\text{=}[\begin{array}{cccc} - & 0 & 0 & 1\\ 0 & - & 0 & 1\\ 0 & 0 & - & 1\\ 0 & 0 & 0 & - \end{array}] \end{array}$$ (29)

Discordance Matrix for Criterion 4 ($C_{w\text{4}}$)


$$\begin{array}{c} D_{\text{4}}\left(A_{wi},A_{wl}\right)\text{=}[\begin{array}{cccc} - & 0 & 0 & 0\\ 0 & - & 0 & 0\\ 0 & 0 & - & 0\\ 0 & 0 & 0 & - \end{array}] \end{array}$$ (30)

Step 9. Determine the outranking degree $\Psi \left(A_{wi},A_{wl}\right)$ by Eq. (17), and we can get:


$$\begin{array}{c} \Psi \left(A_{wi},A_{wl}\right)=[\begin{array}{cccc} - & 0.91 & 1 & 0\\ 0.30 & - & 1 & 0\\ 0 & 0 & - & 0\\ 0 & 0 & 0 & - \end{array}] \end{array}$$ (31)

Step 10. Using Eqs. (18) and (19), we can get the entering flows $\Psi^{\text{+}}\left(A_{wi}\right)$ and leaving flows $\Psi^{\text{-}}\left(A_{wi}\right)$ of each alternative:


$$\begin{array}{c} \Psi^{\text{+}}\left(A_{wi}\right)=\left(\text{1.91,1.30,0,0}\right) \end{array}$$ (32)

$$\begin{array}{c} \Psi^{\text{-}}\left(A_{wi}\right)=\left(\text{0.30,0.91,2,0}\right) \end{array}$$ (33)

Step 11. Using Eq. (20), we can get the net flow of each alternative $\Psi \left(A_{wi}\right)$:


$$\begin{array}{c} \Psi \left(A_{wi}\right)=\left(\text{1.61,0.39,-2.00,0.00}\right) \end{array}$$ (34)

Step 12. Rank all alternatives by the value of $\Psi \left(A_{wi}\right)$, and we can get $A_{w1}≻A_{w2}≻A_{w4}≻A_{w3}$.

Therefore, the four cities in the Tuojiang River Basin—Ziyang, Neijiang, Zigong, and Luzhou—assessed their WRCC using four key indicators: water resources, economy, society, and environment. The assessment ranks the cities in descending order of carrying capacity (or from highest to lowest pressure) as follows: Luzhou, followed by Ziyang, then Zigong and Neijiang

## 4. Discussion

### 4.1 Sensitivity analysis

**Fig 4:**
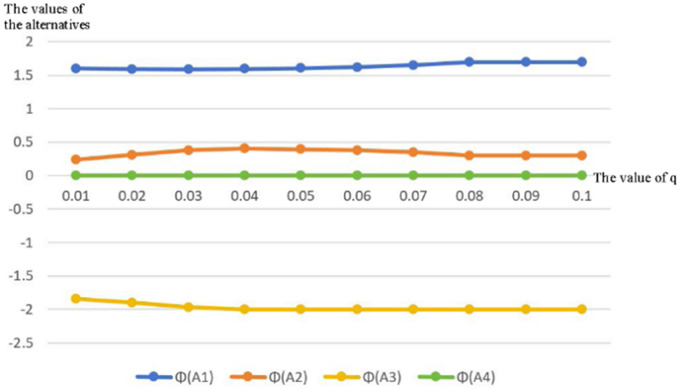
Analyzing the sensitivity of the indifference threshold $q_{i}$ to various values.

**Fig 5:**
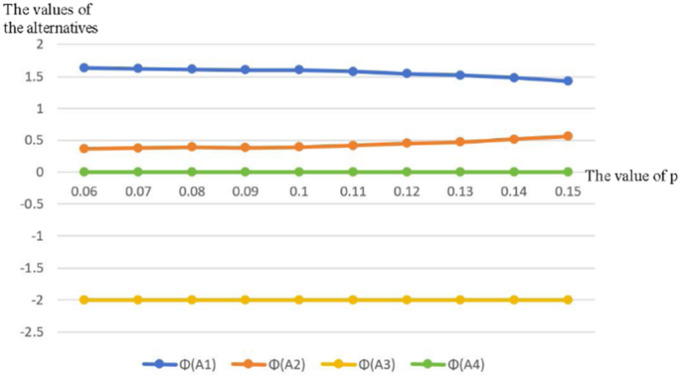
Analyzing the sensitivity of the preference threshold $p_{i}$ to various values.

**Fig 6:**
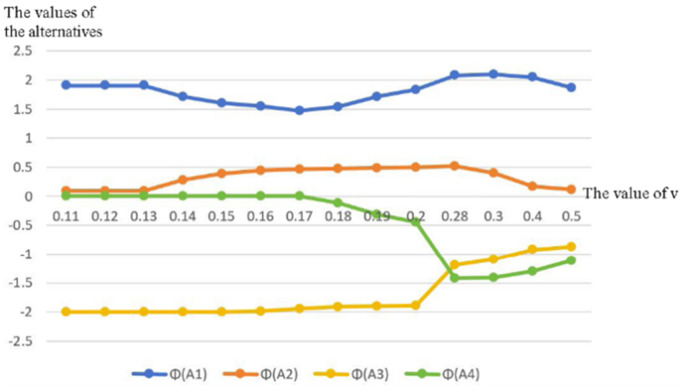
Analyzing the sensitivity of the veto threshold $v_{i}$ to various values.

This section will conduct a sensitivity analysis to explore the impact of utilizing various threshold settings (veto threshold v, preference threshold p, indifference threshold q, and v≥p≥q≥0) on the decision results of the WRCC. To facilitate analysis, we only consider the changes in decision outcomes when v, p and q change separately. Tables 6–8 show the decision-making results. Meanwhile, to highlight the trend of changes in findings, a two-dimensional line chart based on Tables 6–8 is created. In Figs 4–6, the x-coordinate represents the value of $q_{i}$, $p_{i}$, and $v_{i}$ (i=1,2,3,4), while the y-coordinate represents the alternative results in each decision region.

**Table 6:** Sensitivity analysis of the indifference threshold $q_{i}$ to alternative results.

$q_{i}$	$\Psi \left(A_{wi}\right)$	alternative result
(0.01,0.01,0.01,0.01)	(1.60,0.24,-1.85,0.00)	$A_{w1}≻A_{w2}≻A_{w4}≻A_{w3}$
(0.03,0.03,0.03,0.03)	(1.59,0.38,-1.97,0.00)	$A_{w1}≻A_{w2}≻A_{w4}≻A_{w3}$
(0.05,0.05,0.05,0.05)	(1.61,0.39,-2.00,0.00)	$A_{w1}≻A_{w2}≻A_{w4}≻A_{w3}$
(0.07,0.07,0.07,0.07)	(1.65,0.35,-2.00,0.00)	$A_{w1}≻A_{w2}≻A_{w4}≻A_{w3}$
(0.10,0.10,0.10,0.10)	(1.69,0.30,-2.00,0.00)	$A_{w1}≻A_{w2}≻A_{w4}≻A_{w3}$

Note: $p_{i}$ = (0.1, 0.1, 0.1, 0.1); $v_{i}$ = (0.15, 0.15, 0.15, 0.15).

**Table 7:** Sensitivity analysis of the preference threshold $p_{i}$ to alternative results.

$p_{i}$	$\Psi \left(A_{wi}\right)$	alternative result
(0.06,0.06,0.06,0.06)	(1.64,0.36,-2.00,0.00)	$A_{w1}≻A_{w2}≻A_{w4}≻A_{w3}$
(0.08,0.08,0.08,0.08)	(1.61,0.39,-2.00,0.00)	$A_{w1}≻A_{w2}≻A_{w4}≻A_{w3}$
(0.10,0.10,0.10,0.10)	(1.61,0.39,-2.00,0.00)	$A_{w1}≻A_{w2}≻A_{w4}≻A_{w3}$
(0.12,0.12,0.12,0.12)	(1.55,0.45,-2.00,0.00)	$A_{w1}≻A_{w2}≻A_{w4}≻A_{w3}$
(0.14,0.14,0.14,0.14)	(1.48,0.52,-2.00,0.00)	$A_{w1}≻A_{w2}≻A_{w4}≻A_{w3}$

Note: $q_{i}$ = (0.05, 0.05, 0.05, 0.05); $v_{i}$ = (0.15, 0.15, 0.15, 0.15).

**Table 8:** Sensitivity analysis of the veto threshold $v_{i}$ to alternative results.

$v_{i}$	$\Psi \left(A_{wi}\right)$	alternative result
(0.11,0.11,0.11,0.11)	(1.91,0.09,-2.00,0.00)	$A_{w1}≻A_{w2}≻A_{w4}≻A_{w3}$
(0.15,0.15,0.15,0.15)	(1.61,0.39,-2.00,0.00)	$A_{w1}≻A_{w2}≻A_{w4}≻A_{w3}$
(0.20,0.20,0.20,0.20)	(1.83,0.50,-1.89,-0.44)	$A_{w1}≻A_{w2}≻A_{w4}≻A_{w3}$
(0.28,0.28,0.28,0.28)	(2.08,0.52,-1.19,-1.42)	$A_{w1}≻A_{w2}≻A_{w\text{3}}≻A_{w\text{4}}$
(0.30,0.30,0.30,0.30)	(2.10,0.40,-1.09,-1.40)	$A_{w1}≻A_{w2}≻A_{w\text{3}}≻A_{w\text{4}}$
(0.50,0.50,0.50,0.50)	(1.87,0.12,-0.87,-1.11)	$A_{w1}≻A_{w2}≻A_{w\text{3}}≻A_{w\text{4}}$

Note: $q_{i}$ = (0.05, 0.05, 0.05, 0.05); $p_{i}$ = (0.1, 0.1, 0.1, 0.1).

Firstly, q is assessed ten times with a step size of 0.01 from 0.01 to 0.10 to compute the various net flow of each alternative as displayed in Table 6.

The following conclusions are drawn from the results in Tables 6–8 and Figs 4–6.

(1) Under the 10 changes of $q_{i}$ ($q_{\text{1}}=q_{\text{2}}=q_{\text{3}}=q_{\text{4}}$), the orders of four alternatives are consistent and the sorting with $A_{w1}≻A_{w2}≻A_{w4}≻A_{w3}$. With the increase of $q_{i}$, the value of $\Psi \left(A_{w1}\right)$ decreases firstly, then increases, and tends to stabilize when it reaches 1.698; After reaching 0.302, the value of $\Psi \left(A_{w\text{2}}\right)$ tends to settle after first rising and falling; The value of $\Psi \left(A_{w\text{3}}\right)$ continues to drop until it reached −2; $\Psi \left(A_{w\text{4}}\right)$ ’s value stays the same and is entirely zero. No matter how the values of $\Psi \left(A_{wi}\right)$ change, always have $A_{w1}≻A_{w2}≻A_{w4}≻A_{w3}$. This indicates that the net flow of the four cities remains relatively stable when the indifference threshold $q_{i}$ varies, and the ranking of water resource carrying capacity is unchanged: Luzhou ≻ Ziyang ≻ Zigong ≻ Neijiang.
(2) For the different values of $p_{i}$ ($p_{\text{1}}=p_{\text{2}}=p_{\text{3}}=p_{\text{4}}$), the selection of the four alternatives is the same. With the increase of $p_{i}$, $\Psi \left(A_{w1}\right)$ ’s value continues to drop while $\Psi \left(A_{w\text{2}}\right)$ ’s value continues to rise, $\Psi \left(A_{w\text{3}}\right)$ and $\Psi \left(A_{w\text{4}}\right)$ remains unchanged, but still have $A_{w1}≻A_{w2}≻A_{w4}≻A_{w3}$. When the preference threshold p varies, the ranking of WRCC among the four cities remains stable. This demonstrates that the decision is robust to changes in the preference setting, indicating a high level of assessment reliability.
(3) With the increase of $v_{i}$ ($v_{\text{1}}=v_{\text{2}}=v_{\text{3}}=v_{\text{4}}$) value, it continued $A_{w1}≻A_{w2}≻A_{w4}≻A_{w3}$ at first, then $\Psi \left(A_{w\text{3}}\right)$ and $\Psi \left(A_{w\text{4}}\right)$ get closer, when $v_{i}\geq 0.28$, the order of four alternatives has been changed to $A_{w1}≻A_{w2}≻A_{w\text{3}}≻A_{w\text{4}}$. When the veto threshold $v_{i}$ exceeds 0.28, the rankings of Zigong and Neijiang are reversed. This suggests that Zigong exhibits a severe deficiency in a critical criterion within the water resource system. Once DMs tighten the standard to the degree which deemed unacceptable, the veto mechanism is activated. A downgrade in its overall evaluation is caused,and Zigong is ranked below Neijiang.

As a result, the proposed approach can be regarded insensitive to changes in the three-parameter values. In conclusion, the dependability and steadiness of the IF-CPT-PAMSSEM approach for MAGDM problems are demonstrated by the use of sensitivity analyses of WRCC.

### 4.2 Comparative analysis

#### 4.2.1 Compare IF-CPT-PAMSSEM with IF-PAMSSEM.

From the results of Table 9, it is known that the ranking results from the two procedures are identical. The alternatives $A_{w1}$ and $A_{w\text{3}}$ are considered to have the best and worst ecological carrying capacity for water resources respectively in both the IF-CPT-PAMSSEM and the IF-PAMSSEM. Although the ranking results of the four cities from the two techniques are similar, the major difference between IF-CPT-PAMSSEM and IF-PAMSSEM is the dissimilar weighting and value functions. Table 9 clearly shows that their overall dominance differs between the two methodologies. The reason why this difference occurs is that IF-CPT-PAMSSEM’s evaluating information is presented as prospect values based on value functions and attribute weights, while IF-PAMSSEM’s evaluating information may not accurately reflect the psychological perception of DMs. Therefore, the overall dominance between $A_{w\text{1}}$ and $A_{w\text{2}}$ in the IF-CPT-PAMSSEM has changed compared with the IF-PAMSSEM method.

**Table 9:** The results of the two methods.

Methods	$\Psi \left(A_{wi}\right)$	alternative result
IF-CPT-PAMSSEM	(1.61,0.39,-2.00,0.00)	$A_{w1}≻A_{w2}≻A_{w4}≻A_{w3}$
IF-PAMSSEM	(1.79,0.21,-2.00,0.00)	$A_{w1}≻A_{w2}≻A_{w4}≻A_{w3}$

#### 4.2.2 In contrast to certain MAGDM methods in IFSs incorporating CPT.

In this subsection, we use an improved MABAC approach (IF-CPT-MABAC) [50], an improved TODIM method (IF-CPT-TODIM) [29], and an integrated methodology (IF-CPT-VIKOR) [46] to confirm the practicality of our proposed model, respectively. The evaluation results are shown in Table 10.

**Table 10:** Comparative analysis of results across four methods.

	$\Psi \left(A_{w1}\right)$	$\Psi \left(A_{w2}\right)$	$\Psi \left(A_{w3}\right)$	$\Psi \left(A_{w4}\right)$	The final ranking
IF-CPT-MABAC (The total distances)	0.24	0.16	−0.29	0.12	$A_{w1}≻A_{w2}≻A_{w4}≻A_{w3}$
IF-CPT-TODIM (The overall dominance degree)	1.00	0.89	0.00	0.82	$A_{w1}≻A_{w2}≻A_{w4}≻A_{w3}$
IF-CPT-VIKOR (The VIKOR index)	0.00	0.14	0.83	0.61	$A_{w1}≻A_{w2}≻A_{w4}≻A_{w3}$
IF-CPT-PAMSSEM	1.61	0.39	−2.00	0.00	$A_{w1}≻A_{w2}≻A_{w4}≻A_{w3}$

**Fig 7:**
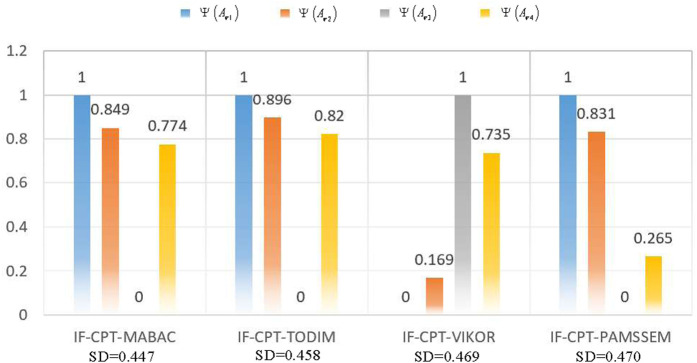
Standard deviation of WRCC evaluation values across four cities under four decision-making frameworks.

(1) The consistent rankings generated by four decision-making approaches highlight the remarkable robustness and reliability of the decision outcomes. Furthermore, this consistency underscores the practicality and scientific rigor of the proposed method in addressing multi-attribute group decision-making problems.
(2) While conventional MCDM methods merely provide a ranking of alternatives in terms of superiority or inferiority, IF-CPT-PAMSSEM is designed to classify each alternative into distinct categories with clearly defined semantics. For instance, in this case, Alternative 1(Luzhou) and Alternative 2(Ziyang) are classified as “acceptable” (positive values), Alternative 4 (Zigong) falls into the “marginal” category (zero value), and Alternative 3(Neijiang) is categorized as “absolutely unacceptable” (significant negative value). This highlights a significant disparity in WRCC among the four cities located in the middle and lower reaches of the Tuojiang River Basin. Luzhou and Ziyang exhibit relatively favorable conditions, Zigong is approaching the critical threshold, while Neijiang faces a severe water resources crisis. This reflects significant imbalances in water resource distribution, utilization efficiency, supply-demand dynamics, and governance within the basin.
(3) Standard Deviation (SD) is a statistical measure that quantifies the degree of dispersion or variability in a dataset. In general, a smaller standard deviation indicates that the data points are closer to the mean and thus more tightly clustered, while a larger standard deviation reflects greater spread or variability around the mean. We first apply min-max normalization independently to the evaluation scores of each decision-making framework reported in Table 10, rescaling all values to the standardized interval [0, 1]. The resulting normalized profiles are: (1.000, 0.849, 0.000, 0.774); (1.000, 0.896, 0.000, 0.820); (0.000, 0.169, 1.000, 0.735); (1.000, 0.831, 0.000, 0.265). For each framework, the standard deviation across its four normalized scores is computed to quantify intra-profile variability—a direct measure of discriminative capacity in multi-criteria evaluation. As shown in Fig 7, the standard deviation of the evaluation values of WRCC for the four cities is 0.447 under the IF-CPT-MABAC framework, 0.458 under IF-CPT-TODIM, 0.469 under IF-CPT-VIKOR, and 0.470 under IF-CPT-PAMSSEM. The IF-CPT-PAMSSEM framework amplifies the differences in assessment values among different cities through its algorithmic design, thereby facilitating a more straightforward and intuitive decision-making process.

### 4.3 Policy implications

The increasing population and rapid socio-economic development have put strains on water resources and threatened regional sustainability. Comprehensive evaluation of water resources is crucial for advancing the sustainable development of national population and development strategies. Changes in rankings directly reflect improvements or deteriorations in key performance areas, enabling policymakers to identify emerging trends, initiate targeted investigations, and adjust policies accordingly. Furthermore, comparative analysis of ranking dynamics allows DMs to extract actionable insights from high-performing cities, providing evidence-based references for strategic planning and policy formulation.

An assessment was conducted on the four cities in the middle and lower reaches of the Tuojiang River Basin, namely Ziyang, Neijiang, Zigong and Luzhou. The WRCC from low to high was Neijiang, Zigong, Ziyang and Luzhou. This ranking clearly reveals the spatial differentiation pattern of water resource pressure in the middle and lower reaches of the Tuojiang River Basin, providing a direct basis for differentiated and precise water resource management policies. For Luzhou, as the city with the strongest carrying capacity, it should undertake more collaborative responsibilities for the entire basin. For Ziyang and Zigong, which are in the middle state, they should strengthen water conservation and carefully assess new water demand, and focus on optimizing the efficiency of agricultural and industrial water use. Neijiang is the most burdened and vulnerable in the WRCC of the basin, and it should priority intervention area for regional water resource management. Any possible industrial layout or urban expansion that may increase water consumption must be subject to the strictest red line constraints. The policy should be tilted towards Neijiang, with a focus on implementing the strictest total and intensity control of water consumption, and vigorously promoting water-saving technology transformation and the utilization of unconventional water sources.

## 5. Conclusions

To effectively address the uncertainties and heterogeneous data inherent in assessing regional WRCC this study proposes an integrated IF-CPT-PAMSSEM model that synergistically combines cumulative prospect theory (CPT), intuitionistic fuzzy sets (IFS), and the PAMSSEM. The framework was rigorously validated through a real-world case study, demonstrating its robustness in supporting DMs with more comprehensive, reliable, and sustainable planning. The following conclusions are derived from this research:

(1) Significant disparities in WRCC among the four cities in the middle and lower reaches of the Tuojiang River Basin. Luzhou and Ziyang demonstrate relatively favorable conditions, whereas Zigong is near a critical threshold, and Neijiang faces a severe water resource crisis. These variations reflect underlying imbalances in water distribution, utilization efficiency, supply-demand dynamics, and governance across the region.
(2) The ranking outcomes generated by the IF-CPT-PAMSSEM model are highly consistent with those produced by three other fundamentally distinct decision-making approaches. This strong agreement confirms the model’s robustness and reliability across diverse methodological frameworks, offering credible support for complex policy decisions.
(3) The IF-CPT-PAMSSEM model produces more distinct and discriminative rankings. Such clarity enhances its utility in guiding resource allocation, prioritizing interventions, and formulating policies, thereby reducing ambiguity in decision-making when alternatives exhibit similar performance scores.

In conclusion, for complex, high-uncertainty, and high-risk decision problems such as WRCC assessment, the IF-CPT-PAMSSEM model represents a theoretically advanced and empirically reliable integrated solution. Nevertheless, the current framework is operated as a static assessment and cannot fully capture the dynamic evolution of water resource systems. Future research should focus on developing a dynamic extension of the PAMSSEM model which is capable of processing multi-temporal data to evaluate spatio-temporal trends in carrying capacity.

## Supporting information

S1 File The dataset underlying the findings presented in this manuscript is provided as a separate file. The data include all relevant experimental measurements, numerical values, and metadata necessary to reproduce the analyses, figures, and conclusions reported in the study. Detailed variable definitions and data structure are described within the file.
(ZIP)
